# Robust fusion for skin lesion segmentation of dermoscopic images

**DOI:** 10.3389/fbioe.2023.1057866

**Published:** 2023-03-20

**Authors:** Qingqing Guo, Xianyong Fang, Linbo Wang, Enming Zhang, Zhengyi Liu

**Affiliations:** ^1^ School of Computer Science and Technology, Anhui University, Hefei, China; ^2^ Islet Pathophysiology, Department of Clinical Science, Lund University Diabetes Centre, Malmö, Sweden

**Keywords:** skin lesion segmentation, transformer, deep learning, robust fusion, gating mechanism

## Abstract

Robust skin lesion segmentation of dermoscopic images is still very difficult. Recent methods often take the combinations of CNN and Transformer for feature abstraction and multi-scale features for further classification. Both types of combination in general rely on some forms of feature fusion. This paper considers these fusions from two novel points of view. For abstraction, Transformer is viewed as the affinity exploration of different patch tokens and can be applied to attend CNN features in multiple scales. Consequently, a new fusion module, the Attention-based Transformer-And-CNN fusion module (ATAC), is proposed. ATAC augments the CNN features with more global contexts. For further classification, adaptively combining the information from multiple scales according to their contributions to object recognition is expected. Accordingly, a new fusion module, the GAting-based Multi-Scale fusion module (GAMS), is also introduced, which adaptively weights the information from multiple scales by the light-weighted gating mechanism. Combining ATAC and GAMS leads to a new encoder-decoder-based framework. In this method, ATAC acts as an encoder block to progressively abstract strong CNN features with rich global contexts attended by long-range relations, while GAMS works as an enhancement of the decoder to generate the discriminative features through adaptive fusion of multi-scale ones. This framework is especially good at lesions of varying sizes and shapes and of low contrasts and its performances are demonstrated with extensive experiments on public skin lesion segmentation datasets.

## 1 Introduction

Skin cancer is listed as one of the fastest-growing cancers in the world (Jemal, 2017) and dermatologists usually identify lesions visually from dermoscopy images captured by dermoscopy. However, manual identification is usually tedious and time-consuming. Therefore, automatic skin lesion segmentation is badly needed in clinical practice, which can assist dermatologists in further analysis.

**FIGURE 1:**
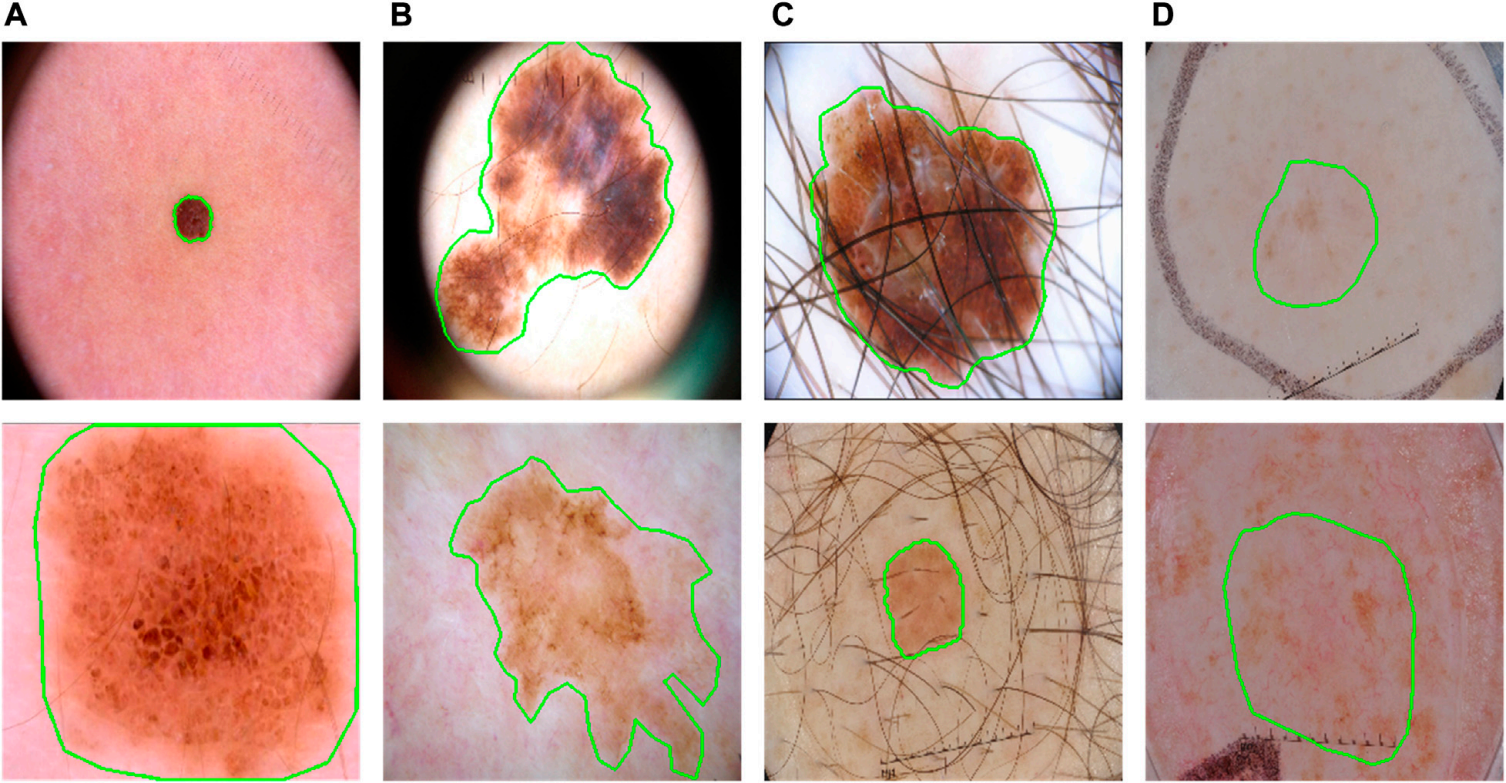
Some typical cases in dermoscopic images for skin lesion segmentation. **(A)** Large variety in sizes; **(B)** large variety in shapes; **(C)** hair occlusion; **(D)** low contrast between lesions and backgrounds.

Skin lesions often have a vast variety of lesion shapes and sizes and are often with low contrasts (Figure 1). It means both global and local contexts are important for an effective feature abstraction, which is also why some methods (Wu et al., 2022; Zhang et al., 2021; Xu et al., 2021; Chen et al., 2021) combine both convolution neural network (CNN) and Transformer (Vaswani et al., 2017): CNN gets features with rich local information while Transformer captures the long-range relationships. They often fuse the two types of feature serially (Chen et al., 2021), or after the last stage of the Transformer branch (Zhang et al., 2021; Xu et al., 2021; Wu et al., 2022).

However, such fusions may not utilize the Transformer effectively. Transformer in principle computes the affinities as attention for long-range relationships. The size and shape variations are significant symbols of lesions (Figure 1), which means a more effective fusion of them can be obtained if applying Transformer as an augmentation to different scales at different encoding stages during the progress of CNN. This progressive boost is very important, especially when facing the low-contrast appearances of lesions.

Therefore, we argue that the better way is to take the Transformer as a progressive attention tool to enhance the long-range information gradually and consequently the feature responses of lesions will be significantly enhanced. Accordingly, a new feature fusion module, the Attention-based Transformer-And-CNN fusion module (ATAC), is proposed. It can fulfill the attention-based fusion progressively in multiple scales which is different from the traditional fusion applied in tandem or after the last stage.

Effectively decoding from the strong features is also important for a successful segmentation, where the fusion of features from different scales is often considered an effective idea. Recent studies show that different scales may have different weights in fusion and features at suboptimal scales may reduce segmentation accuracy (Chen et al., 2016; Shi et al., 2018), *e.g.*, large scales are more important for bigger lesions. Recent methods (Xu et al., 2021; Dai et al., 2022) fuse the multi-scale features with weights computed from several additional convolutions and thus increase the computation complexity.

We prefer a light-weighted scheme to fuse the multi-scale features. Considering that a gating mechanism is effective in filtering the features with fewer parameters, this paper proposes a new multi-scale fusion module, the GAting-based Multi-Scale fusion module (GAMS), to aggregate the multi-scale features adaptively by the weights from gating.

The two fusion modules ATAC and GAMS lead to a new skin lesion segmentation method. Built on the popular U-Net (Ronneberger et al., 2015) structure, it takes ATAC as an encoder block for the effective abstraction of features from both global and local contexts while adopting GAMS as an enhancement to the decoder for robust exploration of the multi-scale features. Experiments show that this method can accurately locate the lesions of different lesion shapes and sizes and low contrasts.

The main contributions can be summarized as follows.

• A novel CNN and Transformer fusion module, ATAC, which takes Transformer features as affinity estimation to attend CNN features for progressively boosting the global contexts.
• A novel multi-scale fusion module, GAMS, which takes weighted contributions from multi-scale features by gating to fuse information from different contexts.
• A new encoder-decoder-based skin lesion segmentation network for single images, which integrates both ATAC and GAMS as the encoder block and decoder enhancement separately and thus can reach robust segmentation of skin lesions without the affection of size and shape variations and low contrasts.

## 2 Related work

### 2.1 Skin lesion segmentation

Traditional skin lesion segmentation methods are mainly based on manually defined traditional features, such as color (Azad et al., 2015; Ashour et al., 2018), shape (Riaz et al., 2018; Silveira et al., 2009), and threshold (Garcia-Arroyo and Garcia-Zapirain, 2019; Pereira et al., 2019), which are not robust and stable. Nowadays many CNN-based methods have been explored for skin lesion segmentation (Yuan and Lo, 2017; Goyal et al., 2020; Yuan et al., 2017; Bi et al., 2017). The popular way is the U-Net (Ronneberger et al., 2015) based idea (Taghanaki et al., 2019; Zhang et al., 2019; Azad et al., 2019; Jha et al., 2020). For example, DoubleU-Net (Jha et al., 2020) uses two U-Net architectures in sequence. Azad et al. (2019) encoded densely connected convolutions into the bottleneck of the encoder-decoder.

More recently, Transformers (Vaswani et al., 2017; Dosovitskiy et al., 2020) have been demonstrated extraordinary capabilities for skin lesion segmentation (Wu et al., 2022; Zhang et al., 2021; Xu et al., 2021; Wang J. et al., 2022, 2021; Reza et al., 2022; Cao et al., 2022). For example, Wang J. et al. (2022), Wang et al. (2021) used boundary information to address ambiguous boundary problems of skin lesion segmentation. Chen et al. (2021) combined CNN and Transformer serially, which may miss some important information required by the successive modules.

Parallel adaption of both CNN and Transformer is also proposed (Wu et al., 2022; Zhang et al., 2021; Xu et al., 2021). Possible fusion methods include concatenation (Wu et al., 2022) and some attention-inspired mechanisms, such as convolution-based attention (Zhang et al., 2021) or direct attention-based supervision (Xu et al., 2021). However, all these fusions happen after the last stages of the Transformer branch and thus may not fully explore the rich contexts from the multi-scale features robustly.

### 2.2 Multi-scale feature aggregation

Some nature image oriented methods (Zhao et al., 2017; Chen et al., 2018; Lin et al., 2017) first extract multi-scale features by pyramid pooling module (PPM), pyramid atrous convolutions, or feature pyramid network (FPN) and then combine these features to predict segmentation results. For skin lesion segmentation, researchers usually first extract multi-scale features by atrous convolution or standard convolution and then fuse them using concatenation or element-wise addition (Zhang et al., 2019; Liu et al., 2019; Cui et al., 2019). Recently, Xu et al. (2021) and Dai et al. (2022) fused multi-scale features by learned weights which are computed by additional convolutions. However, their methods increase the training parameters and consequently the computational complexity.

## 3 Methods

**FIGURE 2:**
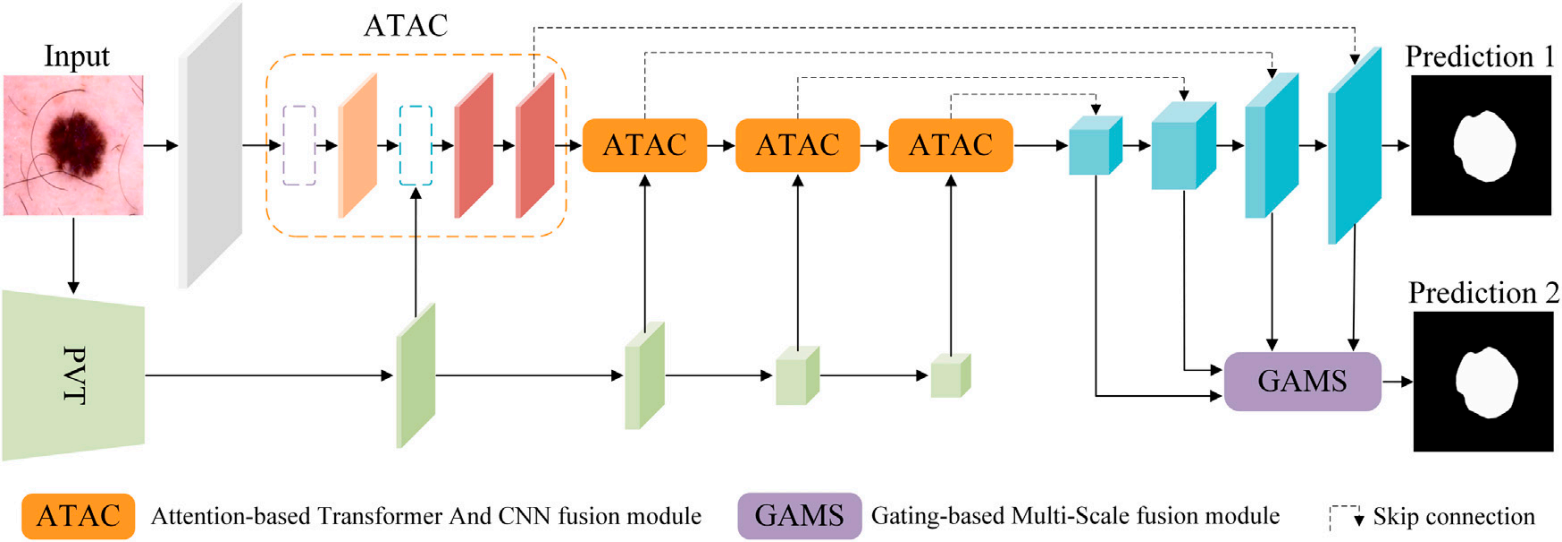
The pipeline of the proposed method. Building on the U-Net and incorporating both CNN and Transformer, it includes two new fusion modules, ATAC and GAMS, into the encoder and decoder respectively for progressively boosting the feature during abstraction and adaptively combining the features for classification respectively. Note: PVT stands for PVT v2, which supplies the Transformer features to ATAC.

Overall, our proposed framework (Figure 2) takes the U-shaped encoder-decoder structure. The encoder adopts ATAC as a building block, which gradually fuses the Transformer and CNN features for feature abstraction. The decoder consists of the normal decoder and its enhancement GAMS. The normal decoder is skipped and connected from the encoder as the typical U-Net, while GAMS takes the features from the decoder for adaptive fusion.

The CNN features of images input to the encoder are attended by the Transformer features stage by stage. Gradually, globally augmented CNN features can be obtained. Then the normal decoder is applied to fulfill the final classification (Prediction 1), while the multi-scale decoder features are also input to GAMS so that effective features aggregated by adaptive fusion are generalized (Prediction 2). The final prediction is based on the results from both predictions.

The four-stage PVT v2 (Wang W. et al., 2022) supplies the Transformer features to ATAC. The normal decoder is made up of up-sampling and two 3 × 3 convolution, as the decoder of UNet (Ronneberger et al., 2015).

Now let’s discuss the details of ATAC and GAMS.

### 3.1 The attention-based transformer-and-CNN fusion module

#### 3.1.1 Why progressive attention?

**FIGURE 3:**
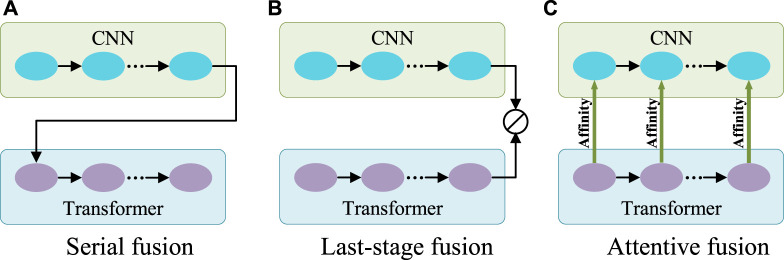
Three different fusion schemes. **(A)** Serial fusion; **(B)** Last-stage Fusion; **(C)** Attentive fusion. Note: 1) CNN and Transformer can change their orders in the serial fusion; 2) ⊘ in **(B)** represents the fusion operation.

Generally, CNN is good at capturing features with rich local details, while Transformer can capture long-range dependence vital to distinguish the target from the background. Therefore, to aggregate information on features from both CNN and Transformer, there are two typical ways of fusion (Figures 3A,B). One can be called serial fusion which treats either CNN or Transformer as neighboring branches and then serially fuses their features. The other can be called last-stage fusion which treats the CNN and Transformer as two parallel branches and fuses their features finally after the last stage of the Transformer branch.

However, serial fusion may not obtain robust features after the second branch because of the possible information loss brought by the filtering effect of the first branch. The last-stage fusion tries to keep all information from the two branches. But the combination after the last stages of the Transformer branch may mess up the information from different scales of the Transformer and cannot effectively utilize it. A more efficient utilization of Transformer features is expected.

Transformer is different from CNN in computing the features. Transformer is configured with the multi-head self-attention for learning the long-range dependencies of image patches. This attention mechanism means that Transformer actually captures the patch affinities globally. The multi-scale Transformer branch supplies rich affinity information from different scales and thus can be used to boost the CNN features progressively as the general attention mechanism for more robust exploration and fusion. Therefore, a novel fusion method called attentive fusion can be obtained (Figure 3C).

Let’s revisit the principle of the multi-head self-attention mechanism in Transformer.

Given a set of *N* tokens $T=\left\{t_{1},t_{2},\ldots ,t_{N}\right\}$ where *t*_*n*_ ∈ *R* ^*d*^ is the *d*-dimension feature vector of the *n*th token (*n* = 1, … , *N*). The multi-head self-attention of Transformer first computes the query *Q*_*i*_, key *K*_*i*_ and value *V*_*i*_ of the *i*-th head of all *p* heads by a linear layer L(⋅) ,

$$Q_{i}=L\left(T\right),K_{i}=L\left(T\right),V_{i}=L\left(T\right).$$ (1)

Then it computes the attention matrix *A*_*i*_ ∈ *R* ^*N*×*N*^, representing affinities between tokens,

$$A_{i}=S\left(\frac{Q_{i}{K_{i}}^{T}}{\sqrt{d_{s}}}\right)$$ (2)

where S represents *Softmax* and *d*_*s*_ is the column dimension of *Q*_*i*_, *K*_*i*_ and *V*_*i*_. After, the feature map of the *i*th head, $H_{i}\in R^{N\times d_{s}}$ , can be computed as

$$H_{i}=A_{i}V_{i}.$$ (3)

The final feature map *L* is obtained by a linear layer after concatenating K all head feature maps,

$$L=L\left(K\left(H_{1},\ldots ,H_{p}\right)\right).$$ (4)

This principle shows that Transformer essentially models the patch affinity. Therefore, its features can be treated as affinities whose global information can be utilized to boost CNN features.

Consequently, to better benefit from Transformer, the proposed attentive fusion is installed as the Transformer attended module ATAC to boost the long-range relations inside the CNN features from different stages and thus progressively dig up significant large-scale contexts. This design fits well with lesions: Their varying sizes and shapes need global contexts to capture, especially considering their possibly low contrasts.

### 3.2 The structure of ATAC

**FIGURE 4:**
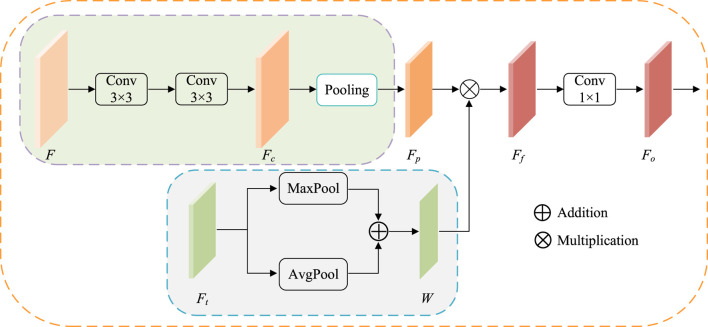
The structure of ATAC. Pooling represents the regular max-pooling operation with the pooling size 2. Maxpool and AvgPool represent max-pooling operation and average-pooling operation along the channel axis respectively.

ATAC is designed as follows (Figure 4). First, the feature maps *F* is 3 × 3 convoluted twice for extracting CNN feature *F*_*c*_, and then regular max-pooling (pooling size is 2) is applied to integrate features as $F_{p}\in R^{c\times h\times w}$ (*c*, *h*, *w* represent channel dimension, height and width of feature map respectively),

$$F_{p}=P\left(C^{3\times 3}\left(C^{3\times 3}\left(F\right)\right)\right),$$ (5)

where $C^{3\times 3}$ and P indicate 3 × 3 convolution and regular max-pooling operation respectively.

At the same time, the corresponding scale Transformer features $F_{t}\in R^{c\times h\times w}$ from the PVT v2 are first mapped to the features $W_{m}\in R^{1\times h\times w}$ and $W_{a}\in R^{1\times h\times w}$ by max-pooling $M_{m}$ and average-pooling $M_{a}$ along the channel axis for integrating information across all channel dimensions, which can be effective in highlighting informative regions. Then they are added to get the fused features $W\in R^{1\times h\times w}$

$$W=M_{a}\left(F_{t}\right)+M_{m}\left(F_{t}\right).$$ (6)

Then, the attention is applied. Here, fused Transformer features *W* are embedded into CNN features *F*_*p*_ by element-wise multiplication to get enhanced CNN features *F*_*f*_. Here, *F*_*p*_ and *W* have the same width and height, so the element-wise multiplication is broadcasted along each channel.

$$F_{f}=W⊙F_{p},$$ (7)

where ⊙ indicates Hadamard product. The output features *F*_*o*_ is finally obtained by 1 × 1 convolution of *F*_*f*_.

### 3.3 The GAting-based multi-scale fusion module

**FIGURE 5:**
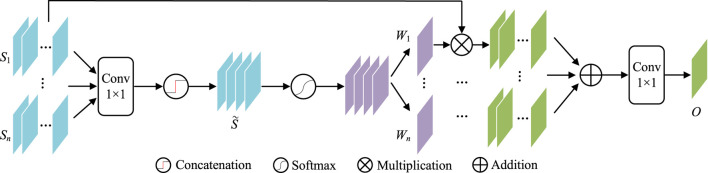
The structure of GAMS.

Contexts from different scales may have different influences on object perception. For example, their large scales are more important for bigger lesions and *vice versa*. It is better to have a weighting scheme to automatically utilize such differences. Considering gating is a very multi-scale filter for such a purpose, this paper introduces GAMS (Figure 5) to improve the feature discrimination.

In GAMS, the input feature maps are first rescaled to the same scale as *S*_*i*_(*i* ∈ {1, … , *n*}) by bilinear upsampling (In our experiment, *n* is set to four according to the four stages of the normal decoder). Then 1 × 1 convolution $C^{1\times 1}$ is applied to reduce the depth of the features to 1. Afterwards, the mapped features are fused by concatenating as $\tilde{S}$ ,

$$\tilde{S}=K\left(C^{1\times 1}\left(S_{1}\right),C^{1\times 1}\left(S_{2}\right),\ldots ,C^{1\times 1}\left(S_{n}\right)\right).$$ (8)

Then, the gating map *W* can be obtained by activation function SoftmaxS ,

$$W=S\left(\tilde{S}\right).$$ (9)

*W* in Eq. 9 is further divided into *W*_1_, *W*_2_, … , *W*_*n*_ as the corresponding weights for the *n* scales. These weights are used to weighted all input features, which are further convoluted by 1 × 1 as the aggregated output features *O*,

$$O=C^{1\times 1}\left(\overset{n}{\underset{i=1}{\sum }}W_{i}⊙S_{i}\right).$$ (10)

### 3.4 Loss function

The overall loss is set to be the weighted average of the losses from both predictions as shown in Figure 2,

$$L_{\mathrm{all}}=\lambda L_{\mathrm{GAMS}}+\left(1-\lambda \right)L_{\mathrm{Normal}},$$ (11)

where: 1) *λ* denotes the weight (*λ* = 0.2 in the experiment); and 2) *L*_*GAMS*_ and *L*_*Normal*_ are the losses from GAMS and the normal decoder respectively. Each loss *L*_*i*_ (*i* ∈ {*GAMS*, *Normal*}) is estimated by the combination of both weighted binary cross-entropy (WBCE) and weighted Intersection over Union (WIOU),

$$L_{i}=l_{\mathrm{IOU}}^{w}\left(p,\hat{p}\right)+l_{\mathrm{BCE}}^{w}\left(p,\hat{p}\right),$$ (12)

where: 1) *p* and $\hat{p}$ indicate the ground truth and prediction respectively; and 2) $l_{\mathrm{IOU}}^{w}\left(\cdot \right)$ and $l_{\mathrm{BCE}}^{w}\left(\cdot \right)$ denote the WBCE and WIOU losses respectively.

## 4 Experiments

### 4.1 Setup

The system is built by PyTorch with a single NVIDIA GeForce GTX 2080Ti GPU. The epoch is 100 and Adam is the optimizer with an initial learning rate of 10^–4^. For PH2, the batch size is set to 8. And for the other three datasets, the batch size is set to 16. All images are re-sized to 256 × 256 as input with various data augmentations, including vertical and horizontal flip, and random rotation.

The proposed method is evaluated on four public skin lesion segmentation datasets, ISIC 2018 (Codella et al., 2019), ISIC 2017 (Codella et al., 2018), ISIC 2016 (Gutman et al., 2016) and PH2 (Mendonça et al., 2013), where the dataset division for ISIC 2017 is the same as the previous study (Reza et al., 2022) with those of the other three following the setting in FAT-Net (Wu et al., 2022). Details of four datasets used in our experiments are described below.

• **ISIC 2016** is provided by the international skin imaging collaboration (ISIC). There are a total of 1279 RGB skin lesions images, of which 900 are used for training and 379 are used for testing.
• **ISIC 2017** is also provided by ISIC, which includes 2000 RGB skin lesion images as the training set with masks for segmentation. We randomly divide the original dataset into a training set, validation set, and testing set in a ratio of 7:1:2.
• **ISIC 2018** is also collected by ISIC, which contains 2594 RGB skin lesions images. Like the ISIC 2017 data set division, we use 1815 samples for the training set, 259 samples for the validation set, and 520 samples for the testing set.
• **PH2** is provided by the dermatology service of hospital Pedro Hispano, Matosinhos, Portugal, which includes 200 RGB skin lesions images. Like ISIC 2017 and ISIC 2018 data set division, we randomly divide them into 140 images as the training set, 20 images as the validation set, and 40 images as the testing set.

The proposed method are compared with some state-of-the-arts methods, including eight CNN-based models (U-Net (Ronneberger et al., 2015), AttU-Net (Schlemper et al., 2019), CPFNet (Feng et al., 2020), DAGAN (Lei et al., 2020), MCGU-Net (Asadi-Aghbolaghi et al., 2020), (Asadi-Aghbolaghi et al., 2020), SBPS (Lee et al., 2020), iFCN (Öztürk and Özkaya, 2020) and CKDNet (Jin et al., 2021)) and three Transformer-based models (TransUNet (Chen et al., 2021), FAT-Net (Wu et al., 2022) and TMUNet Reza et al. (2022)). Among CNN-based models, U-Net and AttU-Net are basic medical image segmentation frameworks. DAGAN, iFCN, and CKDNet are specially designed for skin lesion segmentation. CPFNet, MCGU-Net, and SBPS are excellent segmentation networks in recent years, solving the problems of large size and structure variation and boundary ambiguities, which can be applied to various types of medical images. Among Transformer-based models, TMUNet and FAT-Net fuse CNN and Transformer features at the last stage, while TransUNet fuses CNN and Transformer features serially.

### 4.2 Evaluation metrics

Five widely used metrics are employed to quantitatively evaluate the segmentation performances, including the Sensitivity (SE) (Yerushalmy, 1947), (Yerushalmy, 1947), Specificity (SP) (Yerushalmy, 1947), Intersection over Union (IoU) (Everingham et al., 2015), Dice Similarity Coefficient (DSC) (Dice, 1945), (Dice, 1945)and Accuracy (ACC) (per a la Normalització, 1994). They are defined as:

$$\mathrm{SE}=\frac{TP}{TP+FN},$$ (13)

$$SP=\frac{TN}{TN+FP},$$ (14)

$$IoU=\frac{TP}{TP+FP+FN},$$ (15)

$$DSC=\frac{2\cdot TP}{2\cdot TP+TP+FN},$$ (16)

$$ACC=\frac{TP+FN}{TP+TN+FP+FN},$$ (17)

where: 1) TP (True-Positive) represents the number of pixels that are correctly classified as lesions; 2) TN (True Negative) represents the number of pixels that are correctly classified as backgrounds; 3) FP (False Positive) represents the number of pixels which are falsely classified as lesions; and 4)FN (False Negative) represents the number of pixels which are falsely classified as backgrounds.

### 4.3 Ablation studies

#### 4.3.1 General ablation study

First, the general ablation study for the proposed modules and method for skin lesion segmentation is conducted. U-Net is taken as the baseline. ATAC and GAMS are added to the baseline as different configurations which run on the same environment with the same data augmentations for a fair comparison.

• Baseline The backbone network using U-Net;
• Baseline + ATAC Baseline but replacing its encoder block with ATAC;
• Baseline + GAMS Baseline with the additional GAMS in the decoder;
• Baseline + ATAC + GAMS Our full method.

**FIGURE 6:**
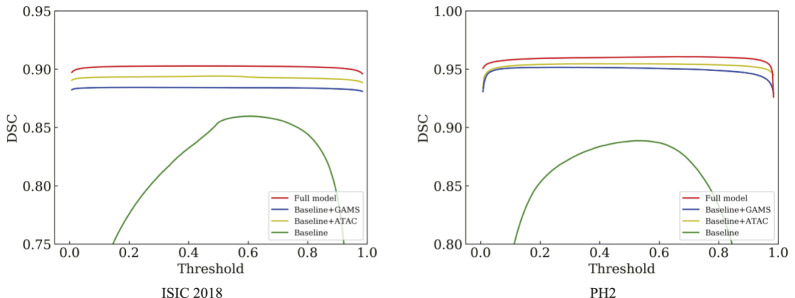
Comparison of the DSC curves under different thresholds on ISIC 2018 and PH2.

Table 1 shows that either ATAC or GAMS improves the performance of the Baseline, demonstrating the effectiveness of each individual component. Our full model further obtains about 0.84% or 2.93% improvements than the model with ATAC or GAMS alone respectively in IoU on ISIC 2018. Similar observations can also be found on PH2. The DSC values under various thresholds are also accumulated (Figure 6), which demonstrates the performance gains by ATAC, GAMS, and the full model over Baseline with the full model being the best among all methods.

**TABLE 1:** Quantitative results for the general ablation study. The best results are shown in bold.

Method	ISIC 2018			PH2		
	ACC	IoU	DSC	ACC	IoU	DSC
Baseline	94.04	77.33	85.45	92.33	84.10	89.36
Baseline + ATAC	96.02	82.89	90.64	97.66	92.40	96.05
Baseline + GAMS	95.50	80.80	89.38	97.22	91.17	95.38
Baseline + ATAC + GAMS	**96.24**	**83.73**	**91.15**	**98.14**	**93.93**	**96.87**

**FIGURE 7:**
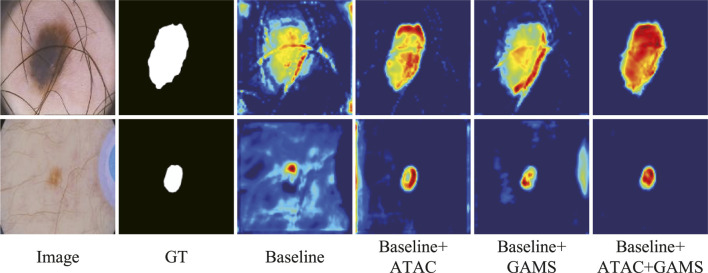
Features from the general ablation study.

The feature maps output by the third stage of the normal decoder in different configurations are also visualized (Figure 7). We randomly selected one-channel feature maps for different configurations, which are uniformly resized to 128 × 128 for better display. The lesions are of different sizes and shapes with the smaller ones in low contrast. ATAC can significantly remove the background distractions because of the global enhancement from Transformer, while GAMS further improves the object responses, especially for the smaller lesion, thanks to its varying weight scheme. Their combination, i.e., the full model, obtains the best result with the strongest maps.

### 4.3.2 Ablation study on the fusion method in ATAC

The ablation study on the fusion method in ATAC is also undertaken (Table 2). Two widely used fusion methods, concatenation and addition, are compared with our proposed attentive fusion, where multiplication operations of ATAC are substituted with concatenation or addition separately. The attentive method achieves the best performances on both ISIC 2018 and PH2 among all methods.

**TABLE 2:** Quantitative results for the ablation study on the fusion method in ATAC. The best results are shown in bold.

Method	ISIC 2018			PH2		
	ACC	IoU	DSC	ACC	IoU	DSC
Concatenation	96.07	83.49	91.00	97.91	93.23	96.50
Addition	96.07	83.27	90.87	97.97	93.40	96.59
Attentive (Ours)	**96.24**	**83.73**	**91.15**	**98.14**	**93.93**	**96.87**

**FIGURE 8:**
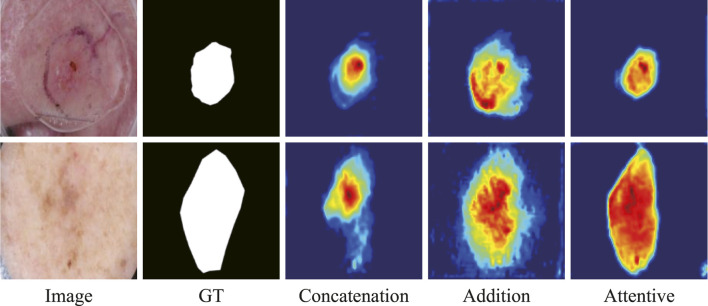
Features from the ablation study on the fusion methods in ATAC.

The features abstracted with different fusion operations are also extracted (Figure 8). The method of feature visualization is the same as in Figure 7. The responses from attentive fusion are stronger and more focused than the other two operations, which also demonstrates the importance of attentive fusion for robust lesion segmentation.

### 4.3.3 Ablation study on the encoder

To further verify the effectiveness of fusion between CNN and Transformer features, an ablation study to compare with only CNN features or only Transformer features in the encoder is also conducted. We replace ATAC with the encoder block of U-Net for only using CNN features. And we replace ATAC with the block of PVT v2 for only using Transformer features. As can be seen in Table 3, our fused encoder achieves the best performance compared with CNN or Transformer encoder alone.

**TABLE 3:** Quantitative results for the ablation study on the encoder. The best results are shown in bold.

Method	ISIC18			PH2		
	ACC	IoU	DSC	ACC	IoU	DSC
CNN encoder	95.50	80.80	89.38	97.22	91.17	95.38
Transformer encoder	96.17	83.61	91.07	97.79	92.99	96.37
Fusion encoder (Ours)	**96.24**	**83.73**	**91.15**	**98.14**	**93.93**	**96.87**

**FIGURE 9:**
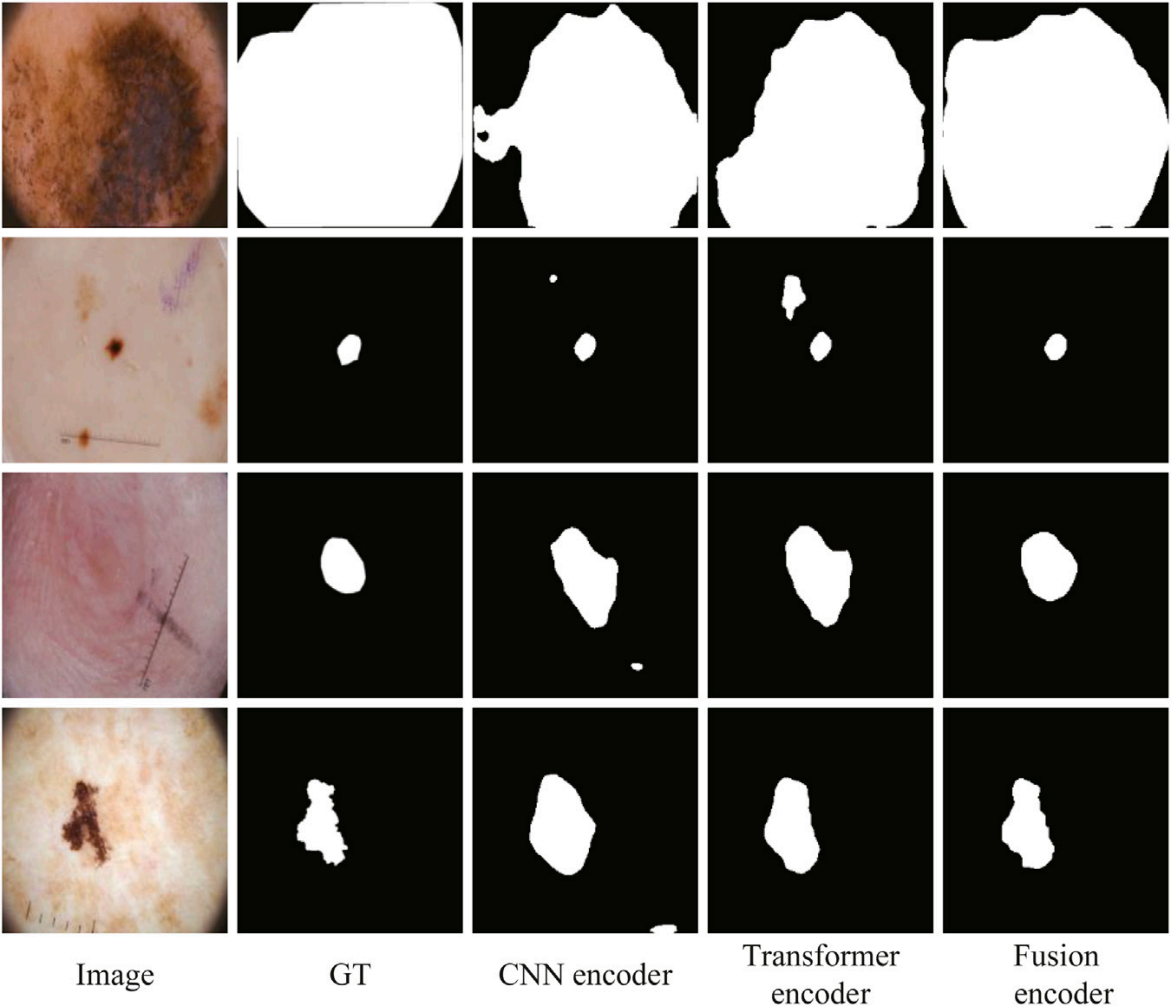
Qualitative comparison for the ablation study on the encoder.

In addition, the segmentation results of some representative images are visualized in Figure 9, including the lesions with various sizes, irregular shapes, and low contrast. The first and second rows show that our fusion encoder yields the best prediction for the smallest or largest lesions. The third row shows the segmentation results for lesions with low contrast. It can be seen that both the CNN encoder and Transformer encoder exhibit over-segmentation, while our fusion encoder achieves the best performance. The last row proves that our fusion encoder segments lesions more accurately for irregularly shaped lesions.

Now, we will discuss the comparisons with the four datasets.

### 4.4 Evaluation on ISIC 2018

#### 4.4.1 Quantitative results

The quantitative results of existing methods are reported by Lin et al. (2022); Wu et al. (2022); Reza et al. (2022) (Table 4). Our method achieves the highest scores in all metrics except SE with a slight decrease.

**TABLE 4:** Statistical comparison of the segmentation results on ISIC 2018. The best results are shown in bold.

Model type	Method	Year	DSC (%)	IoU (%)	ACC (%)	SE (%)	SP (%)
CNN	U-Net	2015	85.45	77.33	94.04	88.00	96.97
	AttU-Net	2019	85.66	77.64	93.76	86.00	98.26
	CPFNet	2020	87.69	79.88	94.96	89.53	96.55
	DAGAN	2020	88.07	81.13	93.24	90.72	95.88
	CKDNet	2021	87.79	80.41	94.92	90.55	97.01
Transformer	TransUNet	2021	88.88	81.85	95.94	90.08	97.89
	FAT-Net	2022	89.03	82.02	95.78	**91.00**	96.99
	TMUNet	2022	90.59	82.80	96.03	90.38	97.46
	**Ours**	2022	**91.15**	**83.73**	**96.24**	88.75	**98.33**

### 4.4.2 Qualitative results

**FIGURE 10:**
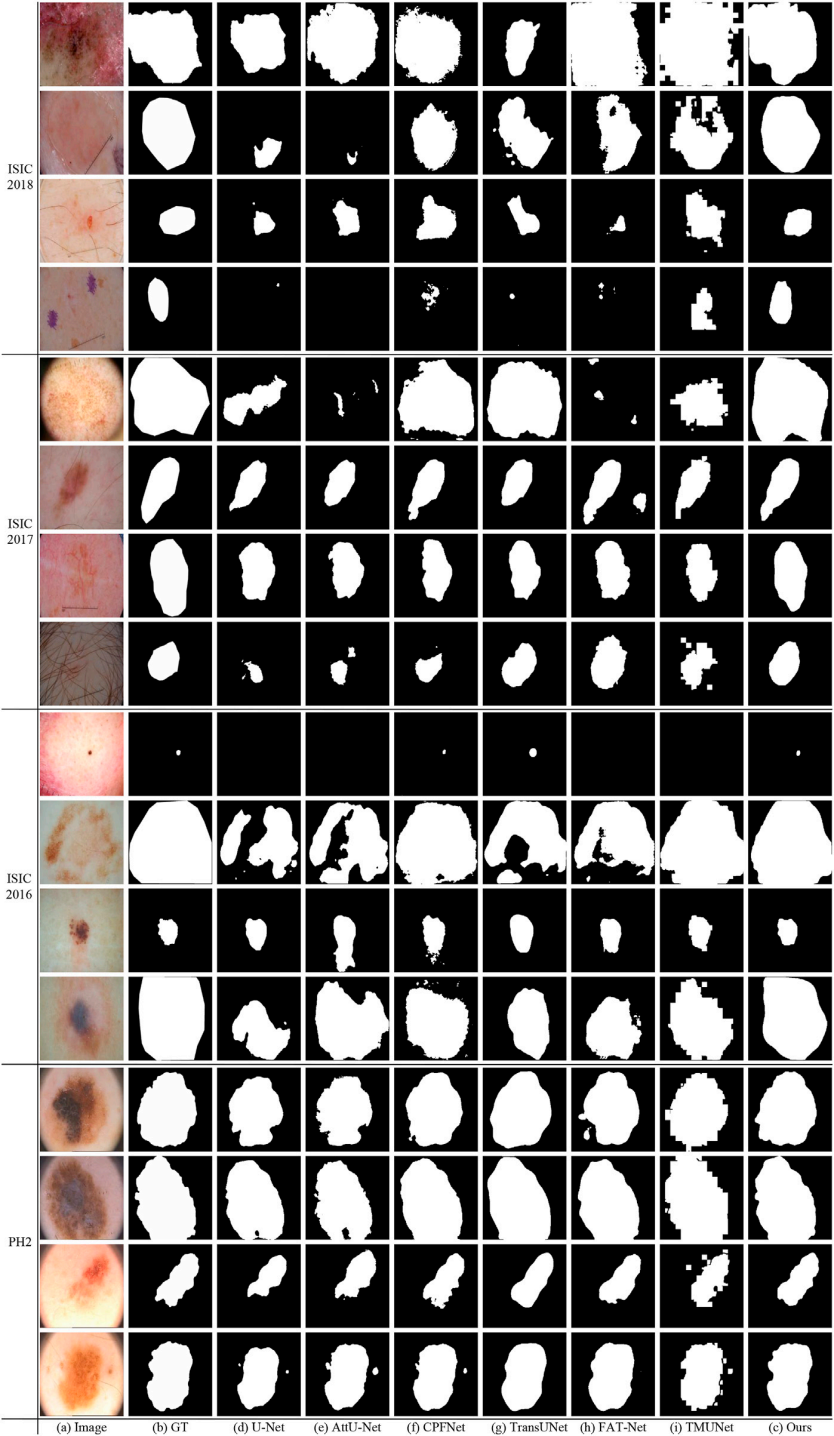
Visual comparison of the segmentation results on ISIC 2018, ISIC 2017, ISIC2016 and PH2.

Figure 10 shows some visualization results of different methods. As can be seen, the lesion has low contrast and ambiguous boundary in the last row of ISIC 2018. The compared methods exhibit under-segmentation. In addition, FAT-Net and TransUNet can struggle to localize a complete lesion because of possible information loss brought by serial fusion and last-stage fusion. Our method benefits from information fusion at different encoder and decoder stages, which can boost feature representation, and thus our method achieves more accurate segmentation results than the compared methods.

### 4.5 Evaluation on ISIC 2017

#### 4.5.1 Quantitative results

The experimental results of DAGAN and MCGU-Net are reported by TMUNet (Reza et al., 2022) with the rest results computed by us according to their released codes (Table 5). Our method also achieves the highest scores in most metrics. In addition, compared with the latest method TMUNet, ours is 0.83%, 1.45%, and 0.44% higher in DSC, IoU, and ACC, respectively.

**TABLE 5:** Statistical comparison of the segmentation results on ISIC 2017. The best results are shown in bold.

Model type	Method	Year	DSC (%)	IoU (%)	ACC (%)	SE (%)	SP (%)
CNN	U-Net	2015	89.64	81.22	96.03	86.22	96.80
	AttU-Net	2019	89.26	80.60	95.96	84.31	98.86
	CPFNet	2020	90.97	83.44	96.60	86.10	**99.21**
	DAGAN	2020	84.25	75.94	93.04	83.63	97.16
	MCGU-Net	2020	89.27	80.62	95.70	85.02	98.55
Transformer	TransUNet	2021	91.54	84.39	96.67	90.54	98.19
	FAT-Net	2022	91.09	83.64	96.54	88.79	98.47
	TMUNet	2022	91.64	84.57	96.60	91.28	97.89
	**Ours**	2022	**92.47**	**86.02**	**97.04**	**91.59**	98.39

### 4.5.2 Qualitative results

Figure 10 shows that our method obtains more accurate results than other methods on ISIC 2017. In the last row of ISIC 2017, the lesion has hair interference. But, apparently, our method is better than other compared methods. It is due to ATAC can effectively utilize the long-range contexts from Transformer, which helps to distinguish different classes.

### 4.6 Evaluation on ISIC 2016

#### 4.6.1 Quantitative results

The quantitative results of existing methods are reported by FAT-Net (Wu et al., 2022) except that those of TransUNet and TMUNet are computed by us according to their released codes (Table 6). Ours again achieves the highest scores in most metrics.

**TABLE 6:** Statistical comparison of the segmentation results on ISIC 2016. The best results are shown in bold.

Model type	Method	Year	DSC (%)	IoU (%)	ACC (%)	SE (%)	SP (%)
CNN	U-Net	2015	88.84	81.84	94.66	90.16	96.56
	AttU-Net	2019	88.75	81.58	94.14	90.31	96.45
	CPFNet	2020	90.23	83.81	95.09	92.11	95.91
	DAGAN	2020	90.85	84.42	95.82	92.28	95.68
	SBPS	2020	90.42	84.34	94.96	92.43	96.13
Transformer	TransUNet	2021	92.12	85.40	95.49	**93.69**	96.19
	FAT-Net	2022	91.59	85.30	96.04	92.59	96.02
	TMUNet	2022	92.20	85.54	95.60	92.32	96.89
	**Ours**	2022	**93.00**	**86.92**	**96.06**	92.80	**97.35**

### 4.6.2 Qualitative results

Figure 10 gives some visual results. As shown in the first and second rows of ISIC 2016, the lesions exhibit a large variation in sizes. But while credit should be given to the fusion of GAMS in different scales of the decoding stage, our method can detect lesions more accurately than other methods, even if they are very small or large.

### 4.7 Evaluation on PH2

#### 4.7.1 Quantitative results

The quantitative results of existing methods are from FAT-Net (Wu et al., 2022), except for CPFNet, TransUNet, CPFNet, and TMUNet, which are computed by us according to their codes (Table 7). Our method again achieves the highest scores for all metrics.

**TABLE 7:** Statistical comparison of the segmentation results on PH2. The best results are shown in bold.

Model type	Method	Year	DSC (%)	IoU (%)	ACC (%)	SE (%)	SP (%)
CNN	U-Net	2015	89.36	84.10	92.33	91.25	95.88
	AttU-Net	2019	90.03	85.82	92.76	92.05	96.40
	CPFNet	2020	95.35	91.12	97.25	95.01	98.19
	DSNet	2020	91.97	87.15	94.82	96.01	96.08
	iFCN	2020	93.21	87.56	96.08	96.13	95.91
Transformer	TransUNet	2021	96.02	92.35	97.62	96.84	97.95
	FAT-Net	2022	94.40	89.62	97.03	94.41	97.41
	TMUNet	2022	92.46	85.97	95.49	93.21	96.45
	**Ours**	2022	**96.87**	**93.93**	**98.14**	**97.17**	**98.54**

### 4.7.2 Qualitative results

As can been seen in PH2 of Figure 10 there are many details around boundary of these lesions. But the boundary obtained by our method is more accurate and closer to the ground truth than other methods. This advantage depends on the strong feature representation capabilities of ATAC.

### 4.8 Failure cases of the proposed method

**FIGURE 11:**
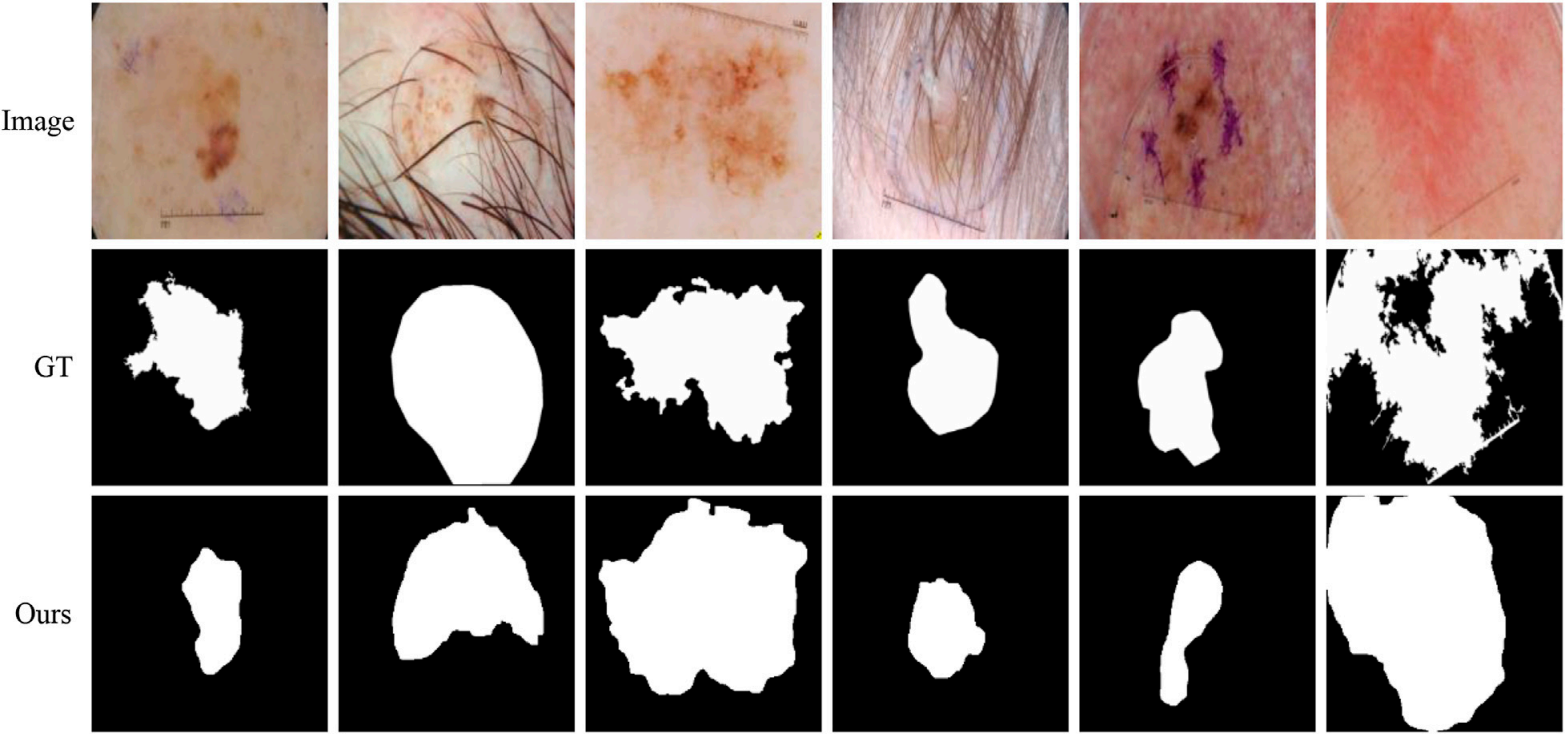
Failure examples of the proposed method.

Although our method is better than the current mainstream segmentation methods, some challenges are still not solved. Figure 11 shows some failure examples. It can be observed that these lesions have very complex boundary regions (see the first, third, and sixth columns) and serious noise interference (see the second, fourth, and fifth column). Our method can basically detect the lesion locations. But in these complex scenes, our method gets poor segmentation results because it is difficult to obtain robust feature representation to distinguish different classes.

## 5 Conclusion

This paper aims at effective fusion policies for robust skin lesion segmentation from dermoscopic images and proposes a new method. Two new fusion modules, ATAC and GAMS, are incorporated in its encoder and decoder for robust feature abstraction and further classification separately. ATAC acts as the encoder block, which takes the Transformer to attend CNN for augmentation of global contexts in different stages. This design makes the abstracted features better fitted for the size and shape of varying lesions, especially when they are in low contrast. GAMS works as an enhancement to the normal decoder, which adaptively weights the features of multiple scales by gating. This module can help obtain features characterized for different objects in low complexity and highly discriminative for robust final inference. Quantitative and qualitative experiments demonstrate the efficacy of the proposed method.

However, ambiguous boundaries of lesions are still challenging. In addition, hair covering the lesions may also distract the model and thus affect the segmentation performances. In the future, we will study those problems and propose more robust methods accordingly.

## DAS

Publicly available datasets were analyzed in this study. For PH2, please find at: https://www.dropbox.com/s/k88qukc20ljnbuo/PH2Dataset.rar; For ISIC 2016, ISIC 2017 and ISIC 2018, please find at: https://challenge.isic-archive.com/data/.
